# Tree Nut Allergy in Children—What Do We Know? —A Review

**DOI:** 10.3390/nu16233978

**Published:** 2024-11-21

**Authors:** Anna Chudoba, Agata Żebrowska, Adam J. Sybilski

**Affiliations:** 1 Clinical Department of Pediatrics and Allergology, National Medical Institute of the Ministry of the Interior and Administration, 02-507 Warsaw, Poland; 2 Department of Pediatrics, Centre of Postgraduate Medical Education, 01-813 Warsaw, Poland

**Keywords:** tree nut allergy, prevalence, diagnosis, avoidance, immunotherapy

## Abstract

Food allergy represents a significant public health concern, with its prevalence increasing in recent decades. Tree nuts are among major allergenic foods, and allergies to them are frequently linked to severe and potentially life-threatening reactions. Data on the prevalence and natural history of tree nut allergy are limited. Primary nut allergy typically presents with rapid-onset IgE-mediated symptoms. Diagnosis can be confirmed by demonstrating a positive skin prick test (SPT), specific IgE (sIgE), or through an oral food challenge. Component-resolved diagnostics (CRD) can identify those with a high risk of anaphylaxis. The main management strategy involves avoiding the culprit allergen and treating symptoms after accidental exposure. New therapeutic options, such as sublingual immunotherapy, oral food immunotherapy, with or without omalizumab, and other monoclonal antibodies, are being investigated to modify tree nut allergy. Tree nut allergy is a lifelong disease with a low likelihood of resolution. The aim of this paper is to present the current data on the prevalence, diagnosis, natural history, and management options for tree nut allergy.

## 1. Introduction

Food allergy is a significant public health problem, with its prevalence increasing in recent decades. It is estimated that 3–10% of children and nearly 10% of adults experience food allergic reactions [1]. Tree nuts (TN) are among the most common food allergens [2].

The most popular tree nuts are hazelnuts, walnuts, cashew nuts, pecans, pistachios, almonds, macadamia nuts, and Brazil nuts [2,3]. It is worth emphasizing that peanuts (PN) are groundnuts, and are classified as a legume [2,4]. Nut allergy is a common trigger of an IgE-mediated reaction. It is characterized by the presence of typical symptoms, ranging from mild to severe and life-threatening, that usually develop within minutes of exposure to the allergen.

Primary allergy is associated with specific IgE antibodies that target major storage proteins (e.g., Cor a 9 for hazelnuts). This type of allergy is characterized by systemic, potentially life-threatening reactions [5]. A cross-reactive allergy between pollen and raw plant foods can lead to pollen–food allergy syndrome (PFAS, formerly known as oral allergy syndrome, OAS). PFAS is characterized by mild symptoms—usually confined to the mouth and pharynx—after ingestion of unprocessed fruits, vegetables, or nuts. The risk of anaphylaxis is relatively low [5,6].

## 2. Prevalence

The worldwide prevalence of TN allergy varies between 0.05 and 4.9%. Studies from the United States indicate that the occurrence of tree nut allergies in children has risen, with self-reported rates increasing from 0.2% in 1997 to 1.1% in 2008 [7,8]. In the majority of studies, the data are derived from self-reported food allergies, which may lead to overestimation [9,10,11]. Systematic reviews were unable to differentiate between the prevalence of IgE-mediated and non-IgE-mediated allergies, as well as the primary allergy and cross-reactive syndromes, due to the limited available data in existing studies [12]. The prevalence of tree nut allergy ranges significantly across different parts of the world, age groups, and diagnostic criteria [10]. According to a recent systematic review and meta-analysis, hazelnut is the most common tree nut allergy in Europe, largely due to its cross-reactivity with birch pollen, while pistachio is the least commonly reported [10,12]. In contrast, the United States has seen a higher prevalence of walnut and cashew allergies, with more recent data indicating that almond and cashew are the predominant triggers of tree nut allergies [10,11,13]. In the United Kingdom, Brazil nut allergy is particularly common [5,10]. In Australia, cashew allergies are reported as the most prevalent, with a clinically confirmed prevalence of up to 3.0% in children aged 6 and 2.3% in those aged 10–14 years old [14,15] Meanwhile, Asian countries typically report lower rates of tree nut allergies. A cross-sectional study in Korea found a 0.32% rate of tree nut allergy among school children [16]. The occurrence of convincing allergy in Singapore was 0.28%, and in the Philippines it was 0.3% [17]. However, there is evidence of an increase in food-induced anaphylaxis, including cases related to tree nuts, particularly in regions like Hong Kong [10].

Tree nut allergy rates vary significantly by age, with children generally showing a higher prevalence compared to adults [9]. The percentage of individuals with sensitization to various nuts rises significantly with age at the time of presentation, from 2% at 0–2 years to 47% at 14 years. This trend may be associated with heightened exposure over time [18].

The frequency of tree nut allergy also differs according to the diagnostic criteria used. For example, in Europe, the point prevalence of self-reported hazelnut allergy is 4%, while the point prevalence of hazelnut allergy confirmed with OFC or DBPCFC is 0.04% [12].

Risk factors contributing to the onset of tree nut allergy have not been studied as thoroughly as those for peanut allergy [5]. A large population-based study reported that eczema at age 2, and asthma and egg allergy at age 4, were significant factors for positive IgE to storage proteins of tree nuts in young adults. Early atopic manifestations may be linked to later sensitization to tree nuts [19].

Homology between tree nut proteins, as well as between tree nuts and peanuts, or seeds, results in a high rate of co-sensitization [10,20]. Confirming the presence of a clinical allergy often requires conducting an oral food challenge [10,21]. An overlap of self-reported symptomatic peanut and TN allergies ranges from 20–60% [1,7,10,14,15,22]. The majority of tree nut-allergic patients are sensitized to other tree nuts [18,23,24,25]; however, clinical relevance is lower. Fleischer et al. reported that 12% of patients allergic to TN reacted to other tree nuts [24]. A retrospective study from the US showed that among patients allergic to tree nuts who were challenged by other tree nuts to which they were sensitized, 24% had positive OFC [21]. In other studies based on clinically diagnosed allergy, co-reactivity to other tree nuts was reported in 30–60% of cases [10,14]. The highest cross-reactivity is observed within the same botanical family, e.g., pecan–walnut (50–100%) and pistachio–cashew (90–100%). Furthermore, between 64.2% and 83.3% of patients allergic to cashew or walnut also reacted to pistachio and pecan, respectively [14,22,23,25,26,27].

TN may also exhibit co-sensitization and co-reactivity with pollens. Individuals primarily allergic to birch pollen, who are sensitized to a PR-10 protein (Bet v1), may present a secondary allergy to tree nuts, resulting in PFAS (homologous PR 10-Cor a 1, Ara h 8, Jug r 5). The frequency of PFAS varies between 4.7% and 20% among patients allergic to pollens [27,28]. Lipid transfer proteins (LTPs) are also associated with tree nut cross-reactivity, with the peach molecule, Pru p 3, as the primary sensitizing allergen [27]. LTP syndrome and PFAS exhibit specific geographical patterns, with LTP syndrome being more prevalent in the Mediterranean region. Interestingly, areas with higher levels of birch pollen show a lower frequency of LTP allergy [27].

## 3. Clinical Manifestation

IgE-mediated food allergy manifests with symptoms affecting one or more systems, including the skin and gastrointestinal tract, as well as the respiratory, cardiovascular, and neurological systems [1].

Recent studies report that TN allergy is responsible for 18–40% of fatalities from anaphylaxis [27,29]. Reactions caused by tree nuts may be more severe than those triggered by peanuts [30]. Identifying those most at risk of severe reactions is essential. A previous severe allergic response is a significant risk factor for future severe events. Asthma is considered to be another risk factor for severe anaphylaxis [5]; however, the results of studies are inconclusive. While poorly controlled asthma definitely worsens the course of the reaction, large epidemiological studies have not confirmed an increased risk of severe clinical presentation of anaphylaxis in patients diagnosed with asthma [31,32]. Most life-threatening reactions occur in teenagers and adults, potentially linked to risk-taking behaviors, such as not avoiding trigger food(s), neglecting to carry an adrenaline auto-injector, or consuming alcohol [5,6].

Tree nut allergies typically develop later than peanut allergies, with peak occurrence estimated at 36 months for tree nuts and 14 months for peanuts [8]. These allergies can also emerge later in adolescence or adulthood. In younger children, a primary allergy is more probable, while older age increases the likelihood of PFAS. Studies have shown that most children with a primary allergy present symptoms during the first known consumption of the specific nut [5,8,20]. In cases of PFAS, individuals typically have a history of consuming the nut without experiencing any symptoms before the onset of PFAS-related reactions [5].

In PFAS, symptoms are usually localized to the oropharynx and mouth, including itchiness of the mouth and throat, and swelling of the lips, tongue, and mouth, which rarely progresses to anaphylaxis [5,28,33]. Additionally, there is often a positive history of other allergic conditions, such as allergic rhinitis and allergic conjunctivitis. Known factors that increase the risk of systemic reaction in PFAS include alcohol, nonsteroidal anti-inflammatory drugs, antacids, proton pump inhibitors, and physical activity [28,34]. PFAS may be diagnosed based on the typical clinical manifestation; however, in cases of suspected nut allergy, the primary allergy to storage proteins (SSP) must be investigated [28]. The differences that help distinguish a primary allergy from PFAS are shown in Table 1 [28]. It is possible that patients with PFAS may not display clinical signs of pollen allergy, but sensitization can be demonstrated through SPT or sIgE evaluations [28]. LTP cross-reaction should be considered if more severe or atypical reactions have occurred after ingestion of raw fruits or vegetables. Lipid transfer proteins are more heat-stable and resistant to proteolytic digestion compared to other pan-allergens, and therefore can cause severe, systemic reactions [28,34].

## 4. Diagnosis

**Figure 1:**
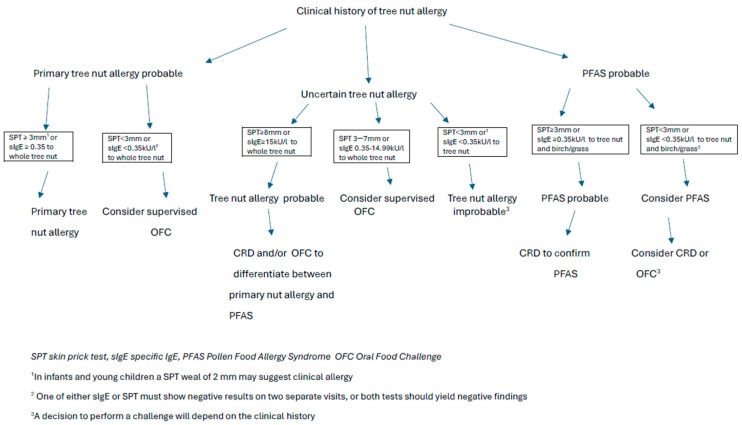
Diagnostic algorithm for tree nut allergy according to BSACI [5] (modified by the author) [5].

A diagnosis of an allergy to tree nuts relies on a mix of clinical history and diagnostic tests, including SPT, sIgE, BAT (Basophil Activation Test), and OFCs [1,5,27]. BAT is not widely available, and is primarily used as a research tool [35,36]. Patient history plays a crucial role in assessing clinical suspicions of allergy and determining appropriate testing. While skin and serum tests indicate sensitization, the patient’ s history is essential for confirming a clinical allergy [1]. A diagnostic algorithm for tree nut allergy, as suggested by BSACI, is presented in Figure 1 [5].

Skin prick tests (SPTs) are conducted using standardized nut extracts, and, sometimes, prick-to-prick testing with a specific nut tree is also used. Clinical history that is consistent with a TN or PN allergy, along with an SPT wheal size ≥3 mm, is generally adequate for diagnosing an allergy [5,37,38]. An SPT cut-off of ≥8 mm for a specific TN is widely regarded as a strong indicator of clinical allergy [5,39]. An SPT < 3 mm is typically effective in ruling out an allergy. However, if the patient presents symptoms suggestive of a tree nut allergy, additional diagnostic tests are typically necessary [5].

There is limited research on the diagnostic accuracy of SPT wheal sizes for tree nut allergies. One study found that an SPT wheal size of ≥8 mm for walnut, hazelnut, and cashew had a positive predictive value (PPV) > 95%. In another study, focusing on hazelnut, skin prick tests with wheal sizes of ≥8 mm and ≥17 mm corresponded to PPVs of 74% and 100%, respectively [5,40].

Conducting SPTs is safe, with systemic reactions being very rare, and the size of the SPT wheal does not correspond to the severity of clinical symptoms [5]. The NUTCRACKER study showed that skin prick tests and the Basophil Activation Test individually were not very effective at differentiating between allergy and tolerance; however, when BAT was combined with SPT, it successfully identified co-allergenicity patterns in patients with sensitization to cashew, pecan, walnut, and pistachio [10,23].

Serum-specific IgE (sIgE) testing for nuts is more accessible than SPTs. An sIgE level of ≥0.35 kU/L is typically considered a positive result, although this cut-off is arbitrary [5]. In research conducted by Fleischer et al., 63% of individuals with a TN sIgE below 2 kU/L successfully completed challenges [9,24]. Additionally, in a retrospective study in the US, it was shown that 89% of patients passed OFC with tree nut sIgE levels <2 kU/L, while 69% of patients passed with levels ≥2 kU/L (mean = 5.12 kU/L) [9,21]. Another observational study reported that an sIgE level of ≥15 kU/L for a specific tree nut had a 95% PPV for clinical allergy [9,41].

BSACI guidelines recommend a diagnostic algorithm for tree nut allergy, suggesting that an SPT ≥ 8 mm or sIgE levels of 15 kU/L or more indicate a likely tree nut allergy. Individuals with ambiguous clinical history or unclear tolerance and SPT sizes between 3 and 7 mm, or sIgE levels from 0.35–14.99 kU/L, may require an oral food challenge [5,10].

Component-resolved diagnostics (CRD) may enhance the accuracy of tree nut allergy diagnoses. While sIgE tests measure IgE levels for the entire food extract, CRD assesses IgE for specific proteins found in the food [9,42]. Depending on the protein family of the allergen, it is possible to identify a primary allergy or cross-reactivity, and to predict the likelihood of severe reactions [4,43]. Seed storage proteins (SSP) and LTP are stable to heat and enzymatic processes, and allergy to them is associated with more severe allergic reactions. In contrast, PR-10 and profilins, which are thermolabile and sensitive to proteolysis, are typically responsible for mild allergic symptoms. 2S albumins, 7S globulins, and 11S globulin/legumins are determinants of primary allergy. LTPs and PR-10 proteins are pan-allergens present in many plant-derived foods, and are makers of cross-reactivity [4,43,44,45]. Tree nut allergens categorized by the protein family are presented in Table 2 [4,43].

## 5. Hazelnut

In Europe, hazelnut is a frequent cause of food-induced systemic allergic reactions and anaphylaxis, particularly among young children [10,46,47]. Thus, an age-based approach to hazelnut allergy proves to be beneficial. Hazelnut sensitization is prevalent among adults, particularly in regions where birch trees are common, as cross-reactivity between PR-10 proteins in birch (Bet v 1) and those in hazelnut (Cor a 1) is a primary factor. In younger children, primary sensitization to SSPs (Cor a 9 and Cor a 14) and LTPs (Cor a 8) is more frequent [46,48,49,50]. Specific IgEs to Cor a 9 and Cor a 14 are linked to a high risk of systemic reactions, and are the most reliable components for diagnosing primary hazelnut allergy [46].

The NPVs for Cor a 9-specific IgE and Cor a 14-specific IgE are strong. Combined testing for their levels has demonstrated a strong NPV (>90%) for primary hazelnut allergy. However, the PPVs for Cor a 9-specific IgE and Cor a 14-specific IgE are low [41,46]. Cor a 8 is a heat-stable LTP that does not cross-react with pollen, and it is known as risk factor for systemic reactions in children from Mediterranean regions [9,51]. IgE to Cor a 8 is correlated with IgE to LTPs found in different foods, particularly in walnuts [46,48,52].

Cor a 1 sIgE demonstrates low PPV, NPV, sensitivity and specificity, making it an unreliable marker for distinguishing primary hazelnut allergy [46,53,54]. Studies revealed that all individuals with PFAS to hazelnut were sensitized to Bet v 1, and over 97% to Cor a 1, while none showed sensitization to Cor a 9 or Cor a 8 [9].

## 6. Cashew and Pistachio

Since pistachios and cashews are part of the same botanical family, cashew allergy is commonly seen in individuals with a pistachio allergy [55]. Cashew and pistachio allergy often trigger severe reactions from minimal exposure. In a retrospective review of medical records for 27 patients with diagnosed cashew allergy, 74% had anaphylaxis after consuming this nut [9,56].

Cashew allergen components contain Ana o 1 (7S globulin), Ana o 2 (11S globulin), and Ana o 3 (2S albumin). Ana o 3 sensitization is the strongest indicator of clinical allergy [9,53]. Brettig et al. reported that Ana o 3 demonstrated the highest accuracy among all CRD tests, and it is considered the most effective method for predicting cashew allergy, based on the inclusion criterion of a positive SPT [36].

Riggioni C. et al. found that SPT with fresh cashew extract showed a high sensitivity of 93% and a high specificity of 92% at the 5 mm cut-off. sIgE to cashew showed a high sensitivity of 94%, with a pooled specificity of 64% at the 1.1 kUA/L cut-off. Ana o 3-sIgE showed a high sensitivity of 96% and a high specificity 94% at the 0.4 kUA/L cut-off [55].

Pistachio proteins Pis v 1 (2S albumin) and Pis v 2 (11S globulin) are similar to Ana o 3 and Ana o 2, respectively, and have been identified as causing sensitization in individuals allergic to pistachios [9,57].

A Turkish prospective study found that SPT wheal sizes for pistachio and cashew were the most reliable indicators for predicting reactivity in challenges. Additionally, the sIgE/total IgE ratio for them was a stronger indicator of positive OFC outcomes than sIgE levels alone. For cashew, the study identified a threshold SPT wheal size of 6.25 mm, similar to previous research, while the cut-off for cashew sIgE was 1.12 kUA/L, below the values reported in the literature. For pistachio, SPT and sIgE thresholds were 7.25 mm and 4.14 kUA/L, respectively. However, unlike prior studies, the cashew and pistachio SPT and sIgE values did not achieve a 95% PPV. This outcome could be attributed to the lower prevalence of pistachio and cashew allergies in children, and the fact that not all children underwent OFC testing [55].

## 7. Walnut and Pecan

Walnut and pecan belong to the same family, and allergies to these nuts frequently occur together, although pecan allergy is somewhat less prevalent. One study revealed that all individuals with a pecan allergy were also allergic to walnut, whereas 9% of those allergic to walnut could tolerate pecan [9,26].

At the moment, eight allergens in walnut (Jug r 1–8) have been formally recognized. Of walnut-allergic patients, 75% develop sIgE against Jug r 1 (2S albumin), which is related to Ana o 3 (2S albumin) [9]. Jug r 2 (vicilin) is found in 60% of the patients and was subsequently classified as a major allergen [4].

Jug r 3 (LTP) was identified in 78.2% of Italian patients with a walnut allergy and may hold greater significance among Mediterranean populations. Notably, this study included patients who experienced both PFAS and systematic reactions [9,58]. Furthermore, Jug r 5 is a protein related to PR-10 [4,58]. A Korean study found that the Jug r 1-sIgE levels were significantly more effective at distinguishing clinical walnut allergy from walnut tolerance in young children than the walnut sIgE levels [59].

A systematic review and meta-analysis indicated that Jug r 1-sIgE demonstrated a sensitivity of 90% at the median cut-off of 0.2 kUA/L. Additionally, four studies on sIgE to walnut met the inclusion criteria for meta-analyses, revealing a pooled sensitivity of 87% and a specificity of 82% [35].

Pecan allergenic components Car i 1 (2S albumin) and Car i 4 (11S legumin) have demonstrated cross-reactivity with Jug r 1 and Jug r 4 [9,60].

For walnut, the component Jug r 1 is the most reliable predictor of walnut allergy. However, Elizur et al. reported that Jug r 4 showed a 95% PPV and proved to be a stronger indicator for identifying patients with dual walnut–pecan allergies [36,61].

## 8. Almond

Almond is reported as an allergy by 9–15% of tree nut-allergic individuals in the US [9,24,57]. Almond sensitization is closely associated with birch sensitization, although this does not always result in clinical symptoms [9,47]. Recognized almond components include Pru du 6 (11S globulin), Pru du 3 (LTP), and Pru du 4 (profilin), though there is limited clinical data on these proteins [9].

In a systematic review and meta-analysis, four studies for sIgE to almond met the inclusion criteria for meta-analysis, with a pooled sensitivity of 72% and 95% specificity, at a median threshold of 3.4 kUA/L [35]. For almond allergy, maximum sensitivity and specificity were ≥90% for almond sIgE, with cut-off medians of 0.8 kUA/L and 7.6 kUA/L, respectively [35].

## 9. BAT

The Basophil Activation Test is an in vitro diagnostic tool that evaluates IgE functionality by assessing its capacity to trigger cell activation and degranulation in response to allergen exposure.

Santos A. et al. in a prospective study, demonstrated that BAT for cashew, almond, sesame, hazelnut, and peanut effectively distinguished allergic from non-allergic children for each respective nut or seed [62]. In this study, patients with an uncertain tree nut (TN) allergy underwent BAT as a second-line diagnostic strategy. This approach reduced the overall need for OFCs by 5–15%, and lowered the rate of positive OFCs by 33–75% (excluding hazelnut), achieving 0% false negatives and a diagnostic accuracy of 96–100%. Additionally, SPT and specific IgE tests in non-allergic tree nut patients (NPV < 95% for all tests) also showed 0% false negatives, and those patients were not assessed using BAT. The BAT, with its high specificity, may complement the high sensitivity of SPT and sIgE [62].

A notable limitation of BAT is the non-responder status, affecting more than 15% of individuals with a food allergy. Another limitation of BAT’s clinical utility is the fact that the test requires fresh blood samples for analysis [36].

## 10. OFC

EAACI guidelines recommend a medically supervised OFC to confirm or exclude food allergy in patients with an uncertain diagnosis despite IgE sensitization tests. Research indicates that fewer than 50% of patients undergoing an oral food challenge experience an allergic reaction. Therefore, OFC is often essential to prevent unnecessary food restrictions, and to avoid potential nutritional deficiencies or food aversion [1,63,64]. For practical purposes, open OFCs are recommended as the standard approach in specialist allergy clinics. They also recommend a double-blind placebo-controlled food challenge (DBPCFC) if the results of an open OFC are inconclusive and for research studies [1]. Challenges may also require consideration of potential co-factors, like exercise, particularly when co-factor-induced reactions cannot be definitively identified through the patient’s history and diagnostic tests [5,65,66]. Elizur et al. have proposed a strategy for conducting OFCs for walnut and pecan nuts, based on the likelihood of an allergic reaction [67]. For individuals with a low probability of walnut/pecan allergy based on clinical assessment, walnut should be tested first; tolerance to walnut suggests an extremely low chance of pecan allergy. On the other hand, in cases of high probability for walnut/pecan allergy, pecan should be challenged first, as a positive result indicates a nearly 100% likelihood of walnut allergy. A similar approach pertains to cashew and pistachio, helping to significantly minimize the need for OFCs [67].

## 11. Management

The management of an IgE-mediated tree nut allergy, as with other food allergies, includes short-term treatment of acute reactions after accidental exposure and a long-term strategy.

Mild primary allergy reactions are usually successfully treated with antihistamines [27,68]. Recognizing the early signs of anaphylaxis and promptly giving adrenaline is extremely important. According to the EAACI, patients with an IgE-mediated allergy and a history of previous anaphylactic reactions triggered by food, with unstable or moderate to severe persistent asthma and systemic mastocytosis, should have easy access to adrenaline auto-injectors and be educated on recognizing and self-managing recurrence of the anaphylactic reactions [27,68,69]. In PFAS, symptoms usually resolve very quickly without any treatment. However, if symptoms persist and cause discomfort, antihistamines should be administered. There is no consensus on prescribing epinephrine for PFAS patients, and recommendations vary among allergists [28,70]. According to the BSACI, epinephrine auto-injectors should be provided for patients with a history of severe reactions [34]. Nowak-Węgrzyn advised prescribing an adrenaline device to all TN-allergic patients, as tree nuts more frequently trigger severe reactions [70].

### 11.1. Dietary Management

A long-term strategy involves avoiding the offending nut. Elimination diets in nut allergy raise a host of questions, one of which is whether all nuts should be avoided, or only those that are clinically relevant [27]. Distinguishing between sensitization and clinically significant co-allergy is challenging and often requires multiple tests and OFCs. Patients and their caregivers should participate in the decision on how restrictive they prefer their diet to be [27].

Eliminating all nuts appears to be the simplest approach, as it reduces the risk of immediate reactions due to accidental exposure or confusion between different nuts. However, it may unnecessarily restrict nuts that could be safely consumed, which could impact nutrition, social interactions, and overall quality of life [5,9,27]. Furthermore, during the period of avoidance, a patient may become sensitized to the nut, and may develop a clinical allergy. The LEAP study (Learning Early About Peanut Allergy) proved that early introduction of peanuts into the diet of infants at high-risk of allergy (patients with egg allergy or atopic dermatitis) reduced peanut allergy occurrence in school children by 86%. It is unknown whether a similar dietary intervention with tree nuts would decrease the risk of tree nut allergy [3,9]. Elizur et al. reported that sensitization levels to tree nuts and peanuts increased significantly, and most of the OFCs performed after the period of avoidance were positive, suggesting that some tree nut allergies developed during the period of the elimination diet [71].

According to the arguments above, a less strict approach to avoidance in tree nut allergy is recommended [68,72,73]. A detailed history and consistent allergy test results, with or without OFC, help to identify the specific allergen that should be avoided. Patients with a primary allergy, as well as those who are allergic to storage proteins or LTPs, which are associated with severe systemic reactions, should eliminate the specific nut from their diet [46]. In the case of PFAS, patients may tolerate the nut when it is boiled, fried, or combined with other food components. However, roasting may increase the allergenicity of the nuts [28,43,70]. Furthermore, patients with PFAS do not need to eliminate potentially cross-reactive nuts or products with PAL [28,46]. Individuals sensitized to LTPs but without a history of reactivity to that food can likely consume it; however, a personalized approach is needed [74]. Co-allergies of walnut–pecan and cashew–pistachio are most commonly reported [14,22,23,25,72]. The NUTCRACKER study found that all individuals allergic to pecan were allergic to walnut, but one third of those allergic to walnut tolerated pecan. Thus, patients reactive to pecan should avoid walnut, but those with a low likelihood of walnut allergy, after passing the OFC for walnut, may consume pecan [67]. Similarly, 100% of individuals allergic to pistachio presented symptoms after cashew ingestion, while 2/3 of those allergic to cashew did not react to pistachio [23]. The same clinical approach as walnut and pecan applies here [67]. As other cross-reactions among tree nuts are less frequent, it may be reasonable to introduce them into the diet. Prior SPT, sIgE evaluation, and OFC may be required [28,44]. Patients who have never ingested a tree nut but are sensitized to them should be considered for OFC [28,68].

It is not recommended to eliminate nuts from the diet if the patient has previously consumed them without any symptoms [68]. After a period of avoidance, reintroduction—even for previously tolerated nuts—may require conducting OFC, especially if sensitization to the tolerated nut is confirmed [68]. Tolerated tree nuts should not be included in diagnostic testing [28,68].

Arguments for and against strict avoidance of tree nuts in tree nut allergy are presented in Table 3 [3,9,75].

Patients should carefully read the ingredient and allergen lists on pre-packaged foods. Some apps can help interpret labels [73]. Global differences in allergen labeling may also lead to accidental exposure [73]. Products with precautionary allergen labeling (PAL) should be avoided by patients with a history of severe reactions [5,73]. Some clinicians suggest that individuals who can tolerate 30 mg of peanut protein may ignore PAL [73]. However, there are reports indicating that risk factors such as sleep deprivation or exercise may lower the threshold for allergic reactions in adult patients with peanut allergy [73,76]. Patients dining out are advised to avoid restaurants where the risk of nut contamination is higher (e.g., Asian restaurants, bakeries), to avoid eating during rush hours, and to inform restaurant staff without delay about their food allergy to ensure that a safe meal option is available. The choice of management strategy should depend on the patient’s age, severity of previous reactions, history of other allergies, the patients’ or caregivers’ level of anxiety, their tendency of risk-taking, and type of lifestyle [5,27,68].

### 11.2. Immunotherapy

Oral immunotherapy (OIT) is based on the long-term, regular administration of a minimum amount of antigen to develop tolerance, desensitization, or to reduce the severity of allergic reactions [2].

A recent systematic review evaluated the use of immunotherapy and biologics in treating tree nut allergy. According to this report, OIT for a single tree nut—hazelnut, walnut, and cashew—achieved success in 41% of hazelnut cases, 89% of walnut cases, and 88% of cashew cases. The efficacy and safety varied depending on the protocol, patient group, and type of tree nut tested [2]. OIT for multiple food allergies, including tree nuts, was studied with and without the addition of omalizumab, and in some studies, antihistamines were used as an adjunctive treatment. Overall, desensitization was reported in 88% of cases, 86% in OIT alone, and 89% in OIT with omalizumab. Hazelnut-OIT showed the lowest effectiveness (70%), while pecan-OIT was the most effective (100%), irrespective of omalizumab use. Achieving maintenance with multiple food OIT required a longer period of time [2]. The same systematic review showed that patients undergoing single-nut-OIT without omalizumab suffered from more severe adverse effects compared to those receiving OIT for multiple foods, with or without omalizumab. However, differences in protocols and patient populations may influence these findings. It was also reported that OIT for one type of nut led to desensitization to other nuts; for instance, walnut-OIT desensitized 53% of individuals allergic to hazelnut, 26% of those allergic to cashew, and 90% of those allergic to pecan.

Erdle et al. investigated the safety and tolerability of OIT for tree nuts in preschool children [77]. Of individuals enrolled in the study, 85.9% were receiving OIT for a single tree nut (cashew, walnut, hazelnut, and macadamia nut), and 14.1% were receiving OIT for multiple tree nuts (two or three types). A total of 95.6% reached the maintenance dose (300 mg of tree nut protein). The results indicated that real-world preschool OIT for tree nuts was safe and comparable to peanut-OIT. Effectiveness will be investigated in future studies [77].

Sublingual immunotherapy (SLIT) was investigated as a treatment of PFAS and LTP syndrome. The efficacy was moderate, with hazelnut-SLIT showing better results than Pru p 3. The safety profile was favorable [2].

Some individuals may experience an improvement in PFAS when undergoing birch subcutaneous immunotherapy (SCIT) or sublingual immunotherapy (SLIT) for rhinoconjunctivitis treatment, but it should not be recommended in the treatment of PFAS alone. Evaluation of the effectiveness and safety of OIT and SLIT for PFAS requires further studies [34].

In contrast to cow’s milk, egg, and peanut allergies, there are fewer recommendations for OIT in tree nut allergies. In a narrative review, researchers summarized practical approaches to OIT in different countries [78]. Among the investigated recommendations, only in Canada is OIT recommended for individuals allergic to all foods, and in Japan, for all patients with IgE-mediated food allergies who have a positive response to OIT, and for whom the allergy is not expected to resolve naturally [78,79,80]. In a recent systematic review and meta-analysis, Riggioni et al. concluded that immunotherapy, with or without omalizumab, appears to be effective and safe for achieving desensitization; however, further studies are needed to investigate the long-term effects [81].

### 11.3. Biologics

Administering omalizumab in food-allergic children, including those with tree nut allergies, raises the threshold for the symptoms’ onset. In the OUtMATCH trial, Wood et al. reported that 41% of patients treated with omalizumab tolerated at least 1000 mg of cashew nut protein in single doses, without dose-limiting symptoms, compared to 3% in the placebo group, which was significant. For walnut, the tolerance was 64% vs. 13%, and for hazelnut it was 65% vs. 14%; *p*-values were not reported, as this was not the aim of the study [82]. The Food and Drug Administration (FDA) has licensed the use of omalizumab as a monotherapy in individuals with IgE-mediated food allergies, including children over 1 year of age in the United States [2]. It has no approval in any other part of the world [2,68,82].

In a recent study, Young et al. investigated sIgE levels of 10 food allergens among children with food allergies and moderate to severe atopic dermatitis who were treated with dupilumab. The estimated percentage decrease in median sIgE levels over one year was 70.9% for hazelnut, 82.5% for cashew nut, 73.8% for almond, 81.2% for walnut, and 81% for pistachio. These findings may suggest the development of tolerance to tree nuts [83]. There are also a few case reports of adult individuals with atopic dermatitis and a co-existing tree nut allergy who achieved tolerance to tree nuts during dupilumab treatment [84,85]. Further studies are needed to confirm the clinical and immunological effects of dupilumab in pediatric patients.

## 12. The Natural History

The natural history of tree nut allergy remains a topic that is not yet fully explored. Tree nut allergy in teenagers and adults rarely resolves [5,6]. Fleischer et al. reported a 9% resolution rate for tree nut allergies. Patients with ongoing allergies to other foods, including other tree nuts and peanuts, as well as those with atopic dermatitis and elevated IgE levels, had lower rates of outgrowing tree nut allergies. Asthma and rhinitis did not influence the likelihood of resolution [24]. Gupta et al. reported resolution in tree nut allergy in 14% of individuals. In the same study, factors associated with a lower likelihood of achieving food tolerance were as follows: races other than White non-Hispanic (both males and females) and for female sex, and multiple food allergies. These factors were mentioned for all allergic foods, generally, including TN [10,13].

There are currently no data on the prevention of tree nut allergy. The LEAP study proved that the early introduction of peanuts into the diet of infants at high-risk of allergy reduced peanut allergy occurrence in school children. It is unknown whether a similar dietary intervention with tree nuts would decrease the risk of tree nut allergy. Additionally, it would be challenging to introduce so many tree nuts at an appropriate dosage for infants [27,86].

## 13. Conclusions

Tree nut allergy is a significant public health concern, with clinical presentation ranging from mild oropharyngeal symptoms to severe, potentially life-threatening reactions. Cross-sensitization and cross-reactivity among tree nuts, between tree nuts and peanuts, and with pollens complicate both diagnosis and the identification of the responsible allergen. While many new diagnostic tools may aid in diagnosis, in predicting reaction severity, the OFC remains the diagnostic gold standard. An elimination diet is a cornerstone of long-term management, with the goal of making it as minimally restrictive as possible. Immunotherapy and biologics hold promise for future treatment. Additionally, data on preventing tree nut allergies are lacking. Further studies are essential to improve the diagnosis, management, and prevention of tree nut allergies.

## Figures and Tables

**Table 1:** Tree nut allergy: distinguishing between primary allergy and PFAS (modified by author).

	Primary Allergy	PFAS
Clinical Manifestation	Systemic, mild to severe reactions	Symptoms limited to mouth and pharynx
Age of Onset	Age < 5 years	Age ≥ 5 years
History of Symptom Onset	First known consumption	After years of tolerance to different forms of nuts
SPT		
Commercial Extract	++	±
Prick-to-Prick with fresh, raw nut	++	++
Extract-pollen	±	++
sIgE		
Whole Nut	+	+
CRD	++ Storage proteins (e.g., Cor a 9, 14)	++ Pollen cross-reactive (e.g., Cor a 1)
Pollen sIgE	±	++

PFAS—pollen–food allergy syndrome; SPT—skin prick test; sIgE—specific IgE; CRD—Component-Resolve Diagnostic. ++—strongly positive, +—positive, ±—variable.

**Table 2:** Tree nut allergens categorized by the protein family [4,43] (modified by the author).

	Family	Seed Storage Proteins			PR-10	LTP	Profilins	Oleosins
Nut		2S Albumin	7S Globulin/Vicilin	11S Globulin/Legumin				
Hazelnut		Cor a 14	Cor a 11	Cor a 9	Cor a 1	Cor a 8	Cor a 2	Cor a 12 Cor a 13 Cor a 15
Walnut		Jug r 1	Jug r 2 Jug r 6	Jug r 4	Jug r 5	Jug r 3	Jug r 7	
Pecan		Car i 1	Car i 2	Car i 4				
Cashew		Ana o 3	Ana o 1	Ana o 2				
Pistachio		Pis v 1	Pis v 3	Pis v 2 Pis v 5				
Macadamia			Mac i 1					
Brazil nut		Ber e 1		Ber e 2				
Amond		Pru du 2S albumin		Pru d 6	Pru du 1		Pru d 4	

LTP—non-specific lipid transfer protein; PR-10—pathogenesis-related protein 10.

**Table 3:** Tree nut allergy: arguments for and against strict avoidance.

Avoidance Option	For	Against
Excluding all tree nuts from the diet, even if clinically tolerant	Simplifies management Reduces risk of cross-contamination of the nuts Reduces risk of misidentification of nuts Decreases number of OFCs to evaluate clinical tolerance	Risk of becoming sensitized and clinically allergic during the period of elimination diet
Not excluding tree nuts that are clinically tolerated	Expands dietary variety Avoids potential risk of becoming sensitized and clinically reactive during elimination diet Limits unnecessary food avoidance	Increases risk of accidental contamination with culprit nut Increases risk of mistaken identification of nuts Increases number of OFCs to evaluate clinical tolerance
